# Two antibacterial and PPARα/γ-agonistic unsaturated keto fatty acids from a coral-associated actinomycete of the genus *Micrococcus*

**DOI:** 10.3762/bjoc.16.29

**Published:** 2020-03-02

**Authors:** Amit Raj Sharma, Enjuro Harunari, Naoya Oku, Nobuyasu Matsuura, Agus Trianto, Yasuhiro Igarashi

**Affiliations:** 1 Biotechnology Research Center and Department of Biotechnology, Toyama Prefectural University, 5180 Kurokawa, Imizu, Toyama 939-0398, Japan; 2 Okayama University of Science, 1-1 Ridaicho, Okayama 700-0005, Japan; 3 Faculty of Fisheries and Marine Sciences, Diponegoro University, Tembalang Campus, St. Prof. Soedarto SH., Semarang 50275, Central Java, Indonesia

**Keywords:** antibacterial, coral, keto fatty acid, *Micrococcus*, PPAR

## Abstract

A pair of geometrically isomeric unsaturated keto fatty acids, (6*E*,8*Z*)- and (6*E*,8*E*)-5-oxo-6,8-tetradecadienoic acids (**1** and **2**), were isolated from the culture broth of an actinomycete of the genus *Micrococcus*, which was associated with a stony coral, *Catalaphyllia* sp. Their chemical structures were elucidated by spectroscopic analysis including NMR and MS, with special assistance of spin system simulation studies for the assignment of an *E* geometry at C8 in **2**. As metabolites of microbes, compounds **1** and **2** are unprecedented in terms of bearing a 2,4-dienone system. Both **1** and **2** showed antibacterial activity against the plant pathogen *Rhizobium radiobacter* and the fish pathogen *Tenacibaculum maritimum*, with a contrasting preference that **1** is more effective to the former strain while **2** is so to the latter. In addition, compounds **1** and **2** displayed agonistic activity against peroxisome proliferator-activated receptors (PPARs) with an isoform specificity towards PPARα and PPARγ.

## Introduction

Marine actinobacteria are considered as a potential source for novel natural products with high structural diversity, unique biological activity, and molecular modes of action beneficial to drug development [1–3]. Actinobacteria in marine environments are mostly found in association with higher organisms, such as fish, sponges, corals, molluscs, ascidians, seaweeds, and mangroves, and have kept attracting attention due to their ability to produce various bioactive compounds [2,4]. Among the isolation sources for marine actinobacteria, substantial amounts of studies were devoted to sponges from which a wide range of actinobacterial species were found to produce intriguing natural products [5]. Corals, another large group of marine invertebrates, also harbor diverse symbiotic or associating microorganisms [6]. However, only a handful of natural products such, as strepchloritides [7], nahuoic acids B–E [8], and pteridic acids C–G [9], were obtained from actinobacteria associated with soft corals. There is no report indeed on natural products from actinobacteria residing in stony corals.

Actinomycetes of the genus *Micrococcus* are Gram-positive, aerobic, and nonmotile cocci. Unlike the majority of actinomycetes, they typically form tetrad clusters but not hyphae [10]. *Micrococcus* is ubiquitous in distribution and, similar to other actinomycetes, marine *Micrococcus* are commonly associated with marine invertebrates, such as sponges and corals [4,11–12]. Distinct classes of natural products have been isolated from sponge-associated *Micrococcus*, including glycosylated glycerolipid [13–14], cyclic peptide [15], xanthone glycoside [16], and halogenated diphenyl ether [14]. Until now, however, no natural products are known from coral-associated *Micrococcus*.

As a part of our ongoing screening program to discover new natural products from coral-associated bacteria, we have recently reported a catecholate siderophore, labrenzbactin, from an alphaproteobacterium *Labrenzia* [17] and an unsaturated fatty acid with unique methylation pattern from a gammaproteobacterium *Microbulbifer* [18]. Herein, we report the fermentation, isolation, structure determination, and bioactivity of two new keto fatty acids, (6*E*,8*Z*)-5-oxo-6,8-tetradecadienoic acid (**1**) and its (6*E*,8*E*)-isomer **2**, from a coral-associated actinomycete *Micrococcus* sp. C5-9.

## Results and Discussion

**Figure 1:**
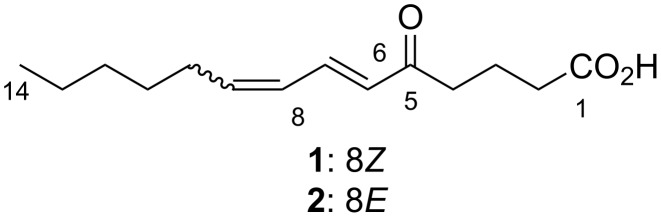
Structures of (6*E*,8*Z*)- and (6*E*,8*E*)-5-oxo-6,8-tetradecadienoic acids (**1** and **2**).

**Figure 2:**
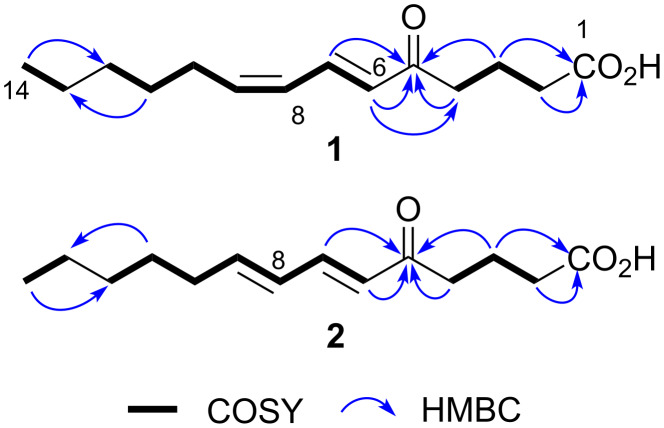
COSY and key HMBC correlations for **1** and **2**.

The producing strain C5-9 was obtained from stony coral *Catalaphyllia* sp. and identified as a member of the genus *Micrococcus* by 16S rRNA gene sequence analysis. The HPLC–UV analysis of the fermentation broth of strain C5-9 indicated the presence of metabolites showing UV absorption around 275 nm. A large-scale shaking culture (2.9 L) was carried out in A16 seawater medium at 30 °C for five days to obtain adequate amounts of compounds for structure determination and bioassays. The fermentation broth was extracted with 1-butanol, and the extract was fractionated by solvent/solvent partitioning. Following ODS column chromatography and isocratic reversed-phase preparative HPLC, **1** (3.7 mg) and **2** (2.4 mg) were isolated (Figure 1).

(6*E*,8*Z*)-5-Oxo-6,8-tetradecadienoic acid (**1**) was obtained as a pale yellow amorphous solid. The molecular formula was determined to be C_14_H_22_O_3_ on the basis of its NMR and HRESIMS-TOF data (*m*/*z* 261.1453 [M + Na]^+^; calcd for C_14_H_22_O_3_Na, 261.1461). The UV spectrum of **1** in methanol exhibited an absorption maximum at 277 nm. The IR absorption bands at 1708 and 2800–3400 cm^−1^ were suggestive of the carbonyl and hydroxy functionalities. The ^13^C NMR and DEPT spectra of **1** (Table 1) displayed 14 carbon signals, including one methyl, seven sp^3^ methylene, four sp^2^ methine (δ_C_ 143.1, 137.5, 129.1, and 126.8), one carboxy (δ_C_ 178.2), and one deshielded aldehyde or keto carbon signal (δ_C_ 199.8). Four degrees of unsaturation indicated by ^13^C signals were consistent with the number calculated from the molecular formula, which indicated that **1** had a linear structure. The ^1^H NMR spectrum showed characteristic resonances for a terminal methyl group at δ_H_ 0.89 (3H, t) in the shielded region and for multiple methylene signals, suggesting the presence of an alkyl chain. COSY analysis established three spin systems, one from H2 to H4, a seven-carbon fragment from H6 to H12, and an ethyl fragment H13/H14. These partial structures were joined into one linear structure by HMBC correlations from H3, H4, H6, and H7 to C5, H14 to C12, and H11 to C13. Then, a correlation from H2 and H3 to C1 connected the carboxy group at C2 to complete the structure of **1** (Figure 2). The geometry of the double bonds was *E* at C6 and *Z* at C8 based on the coupling constants *J*_H6,H7_ = 15.3 Hz and *J*_H8,H9_ = 10.8 Hz, respectively.

**Table 1:** ^1^H and ^13^C NMR data for compounds **1** and **2** in CDCl_3_.

	**1**			**2**		
	
position	δ_C_^a^	δ_H_ mult (*J* in Hz)^b^	HMBC^b,c^	δ_C_^a^	δ_H_ mult (*J* in Hz)^b^	HMBC^b,c^

1	178.2, C			177.7, C		
2	32.9, CH_2_	2.44, m	1, 3, 4	32.9, CH_2_	2.42, m	1, 3, 4
3	19.1, CH_2_	1.97, m	1, 2, 4, 5	19.1, CH_2_	1.96, quint (7.0)	1, 2, 4, 5
4	39.6, CH_2_	2.66, t (6.6)	2, 3, 5	39.0, CH_2_	2.65, t (7.1)	2, 3, 5
5	199.8, C			199.8, C		
6	129.1, CH	6.15, d (15.3)	4, 5, 8	127.6, CH	6.07, d (15.6)	4, 5, 8
7	137.5, CH	7.51, dd (15.3, 11.7)	5, 6, 8, 9	143.5, CH	7.15, dd (15.6, 9.5)	5, 8, 9
8	126.8, CH	6.10, dd (11.7, 10.8)	5, 6, 7, 10	128.7, CH	6.15, dd (15.6, 9.5)^d^	6, 7, 10
9	143.1, CH	5.92, dt (10.8, 7.9)	7, 8, 10, 11	146.2, CH	6.18, dt (15.6, 6.3)^d^	7, 10, 11
10	28.3^e^, CH_2_	2.31, q (7.5)	8, 9, 11, 12	33.1, CH_2_	2.18, dt (6.3, 7.2)	8, 9, 11, 12
11	29.0^e^, CH_2_	1.43, quint (7.1)	9, 10, 12, 13	28.3, CH_2_	1.43, quint (7.2)	10, 12, 13
12	31.4, CH_2_	1.32^f^, m	10, 11, 13	31.4, CH_2_	1.29^f^, m	11, 13
13	22.5, CH_2_	1.30^f^, m	12, 14	22.4, CH_2_	1.31^f^, m	12
14	14.0, CH_3_	0.89, t (6.8)	12, 13	14.0, CH_3_	0.89, t (6.9)	12, 13

^a^Recorded at 125 MHz (reference δ_C_ 77.0). ^b^Recorded at 500 MHz (reference δ_H_ 7.26). ^c^HMBC correlations are from proton(s) stated to the indicated carbon atom. ^d^Determined by NMR simulations. ^e^Assignment interchangeable. ^f^Overlapping signals.

**Figure 3:**
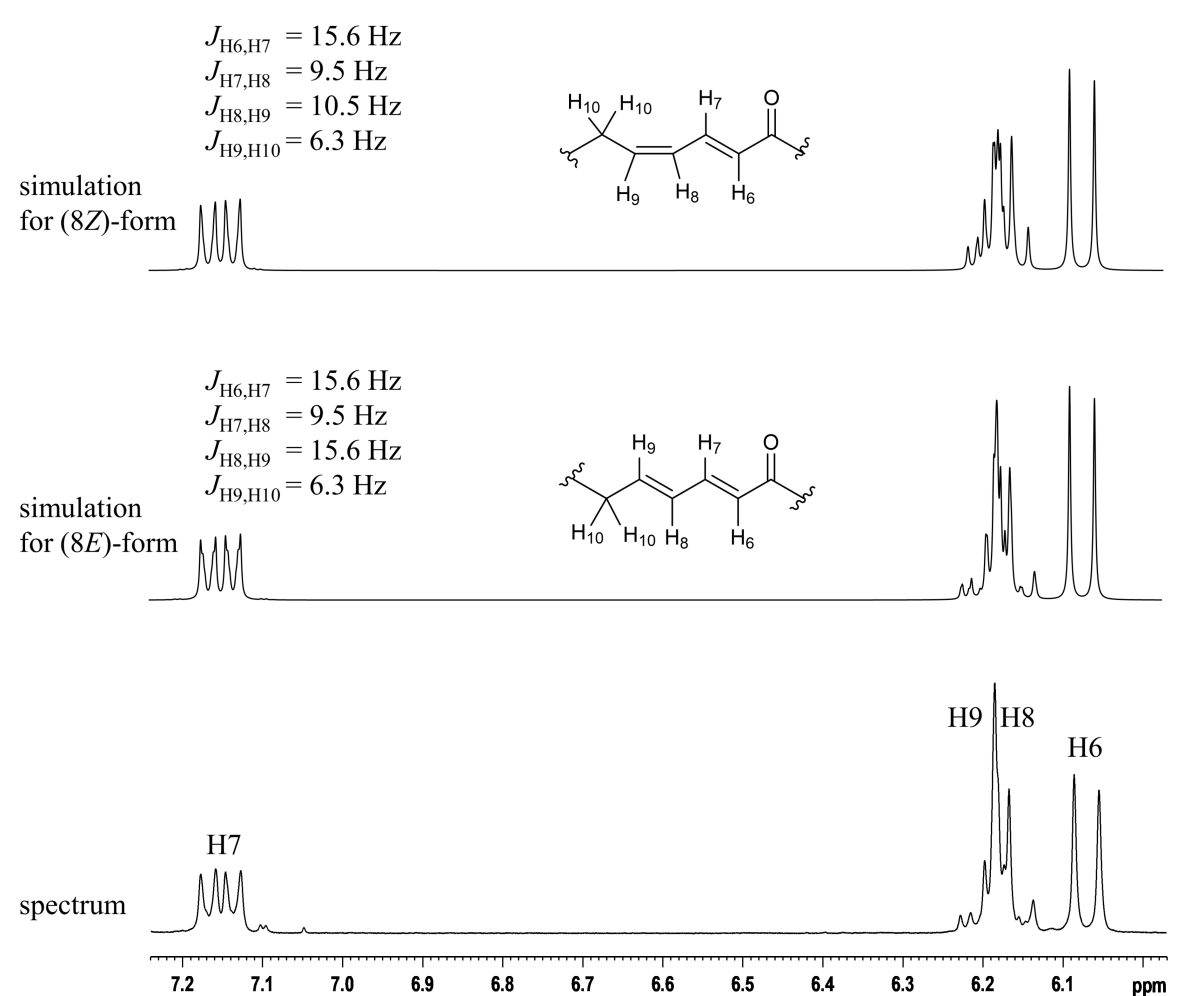
Spin system simulation for the C8–C9 double bond of **2**.

The molecular formula of **2** was also determined to be C_14_H_22_O_3_ on the basis of its NMR and HRESIMS-TOF data (*m*/*z* 261.1458 [M + Na]^+^; calcd for C_14_H_22_O_3_Na, 261.1461). The ^1^H and ^13^C NMR spectra of **2** displayed similar features to those of **1** except five ^1^H/^13^C resonances from C6 to C10, which indicated the structural difference between **1** and **2** to be in the double bond geometries. In fact, the composition of 14 carbon signals, the carbon connectivity, and the sites of functional groups in **2** proved to be completely the same as those in **1** by the interpretation of ^13^C, DEPT, COSY, and HMBC correlations (Figure 2). While an *E* configuration at C6 was evident from the coupling constant *J*_H6,H7_ = 15.6 Hz, *J*_H8,H9_ was unable to be read from the multiplicity pattern of H8 and H9 due to the intense second-order effects caused by a signal overlap of these resonances (δ_H_ 6.15 and 6.18, respectively). Although the *E* geometry at C8 was circumstantially obvious and supported by the deshielded allylic carbon atom C10 (δ_C_ 33.1 for **2** vs 28.3 for **1**), a decisive evidence was acquired from spin system simulations using the software ‘nmrpeak’ [19], which gave the best match to the experimentally obtained ^1^H NMR spectrum with the setting of ^3^*J*_H8,H9_ = 15.6 Hz and ^3^*J*_H7,H8_ = 9.5 Hz (Figure 3). Thus, the C8 geometry was unambiguously determined to be *E*.

**Figure 4:**
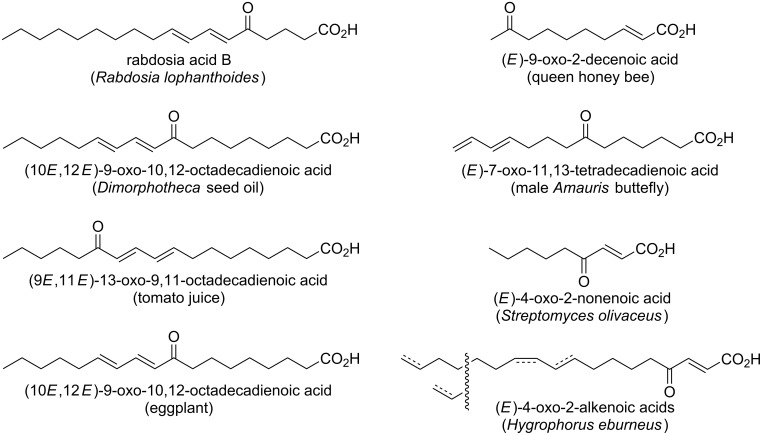
Natural keto fatty acids of various origins.

α-Keto fatty acids are characterized by the presence of a keto group at the α-position of a carboxylic acid moiety. They are present in all living cells and play crucial roles in biological systems as they are involved in the Krebs cycle and glycolysis [20]. In contrast, keto fatty acids bearing a keto group in the middle of the carbon chain are relatively limited in their distribution in nature. Many of such natural keto fatty acids were found in plants, mainly as a constituent of seed oil [21–29]. Among them, rabdosia acids [30] and (10*E*,12*E*)-9-oxo-10,12-octadecadienoic acid [31] are the plant keto fatty acids containing the dienone moiety with *trans*,*trans*-configuration, but congeners with *trans*,*cis*-configuration have not been found in nature until the present work (Figure 4). Some of the keto fatty acids of plant origin were shown to exhibit pharmaceutically important activity. (9*E*,11*E*)-13-Oxooctadecadienoic acid is a PPARα activator found in tomato juice. This keto fatty acid decreases plasma and hepatic triglyceride in obese diabetic mice by activating PPARα transcription [32]. (10*E*,12*E*)-9-Oxooctadecadienoic acid isolated from eggplant calyx induces apoptosis in human ovarian cancer cells, leading to cell death [33]. One example of a keto fatty acid from the animal kingdom is (*E*)-9-oxo-2-decenoic acid, a sex pheromone found in royal jelly. Queen honey bees use this fatty acid to control the activity of worker bees [34]. (*E*)-7-Oxo-11,13-tetradecadienoic acid is another example of insect origin, identified from hair pencils of male *Amauris* butterflies (*Amauris albimaculata*), which is supposed to be a precursor material for the butterfly pheromone [35]. Furthermore, 4-oxo-2-alkenoic fatty acids were characterized as antimicrobial metabolites from an actinomycete [36] and a basidiomycete fungus [37]. In addition, long-chain saturated fatty acids possessing a keto group were detected in the solvent extract of *Legionella* by GC–MS analysis [38]. Fatty acid components in fresh water-derived *Micrococcus* species were comprehensively analyzed [39], but keto fatty acids like compounds **1** and **2**, bearing a dienone system, are unprecedented as microbial metabolites.

**Figure 5:**
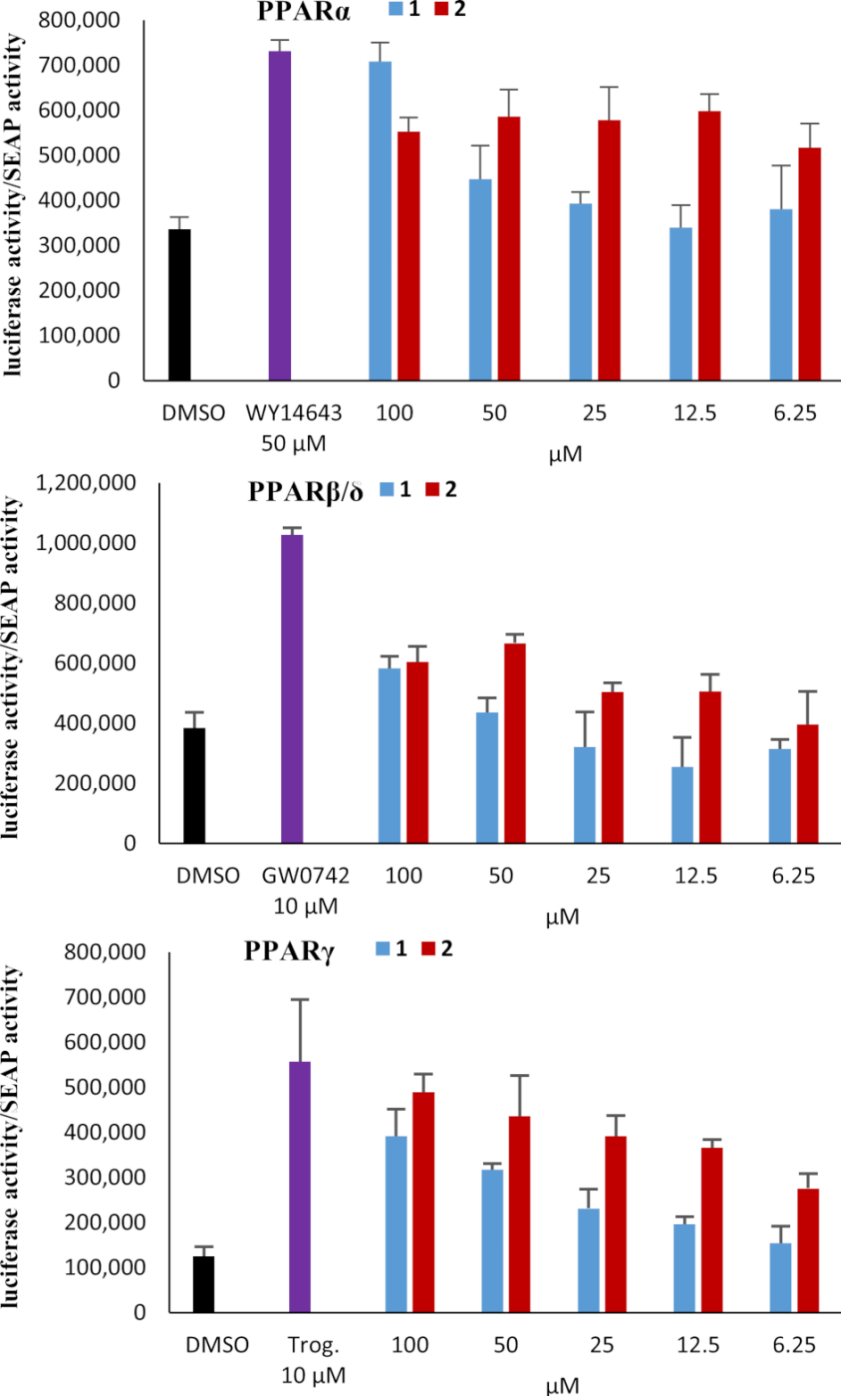
PPAR activation by **1** and **2**.

Compounds **1** and **2** inhibited the growth of *Tenacibaculum maritimum* NBRC16015, a causative agent of skin infection of marine fish [40], and *Rhizobium radiobacter* NBRC14554, a causative agent of crown gall disease of plants [41]. The MIC values for **1** against *T. maritimum* and *R. radiobacter* were 50 and 6.2 µg/mL, respectively, while **2** was more potent against *T. maritimum*, with a MIC of 12.5 µg/mL, and less against *R. radiobacter*, with a MIC of 50 µg/mL, presenting an interesting contrast. No appreciable antimicrobial activity was observed for both compounds against bacterial strains of *Micrococcus luteus* ATCC9341, *Staphylococcus aureus* FDA209P JC-1, and *Escherichia coli* NIHJ JC-2 and yeast strains of *Candida albicans* NBRC0197 and *Saccharomyces cerevisiae* S100, nor cytotoxicity against murine leukemia P388 cells at 100 µM. Additionally, compounds **1** and **2** were evaluated for agonist activity to peroxisome proliferator-activated receptors (PPARs) because similar oxo fatty acids are known to act as PPAR agonists [42]. PPARs are ligand-activated transcription factors playing key roles in lipid and carbohydrate metabolism [43–44]. PPARα upregulates lipid uptake and β-oxidation of fatty acids, whereas PPARγ promotes adipocyte differentiation and adipokine production in adipose tissues to improve insulin sensitivity in diabetic patients [45–47]. Owing to these physiological functions in energy metabolism, PPARs are the molecular targets of metabolic disorders [48]. To assess the PPAR isoform specificity of **1** and **2**, three reporter cell lines expressing luciferase genes in response to PPARα, PPARβ/δ, and PPARγ agonists were used [49]. The agonist activity was determined as a relative potency to the positive controls, WY14643 for PPARα, GW0742 for PPARβ/δ, and troglitazone for PPARγ. Both **1** and **2** induced activations of PPARα and PPARγ transcription but were not effective against PPARβ/δ (Figure 5). Compared to the activity at 12.5 μM, slight increases of PPARα and β/δ agonist activities were observed for **1** at the lower concentration of 6.25 μM, but the activity at 6.25 μM was almost equivalent to the activity level of the vehicle DMSO. We thus considered that **1** had no significant activity at 6.25 μM. Overall, **1** was lesser potent than **2**, indicating that the geometry at C8 may play a crucial role in the binding to PPARs.

## Conclusion

In summary, UV-chemical screening of the coral-associated bacterium *Micrococcus* sp. C5-9 led to the discovery of two new unsaturated keto fatty acids, (6*E*,8*Z*)-5-oxo-6,8-tetradecadienoic acid (**1**) and its (6*E*,8*E*)-isomer **2**. Compounds **1** and **2** showed selective antibacterial activity against the plant pathogen *R. radiobacter* and the fish pathogen *T. maritimum*, respectively. In addition, both **1** and **2** displayed agonistic activity against PPARα and PPARγ.

## Experimental

### General experimental procedure

UV and IR spectra were recorded on a Shimadzu UV-1800 and a Perkin-Elmer Spectrum 100 spectrophotometer, respectively. NMR spectra were obtained on a Bruker AVANCE 500 spectrometer in CDCl_3_ using the signals of the residual solvent (δ_H_ 7.26) and (δ_C_ 77.0) as internal standards. HRESIMS-TOF was recorded on a Bruker micrOTOF focus.

### Microorganism

Strain C5-9 was collected from a stony coral, *Catalaphyllia* sp., obtained from an aquarium vendor in Osaka, Japan. A piece of the coral specimen (ca. 1 g) was surface-sterilized by washing with 70% ethanol, followed by rinsing with sterile natural seawater. The coral piece was homogenized by mortar and pestle with an equal volume of sterile natural seawater (1 mL), and the resulting suspension was serially diluted tenfold to 10^−5^. One-hundred μL aliquots of each dilution were spread onto Marine Agar 2216 (Difco), and the agar plates were cultivated at 23 °C for two days. Single colonies thus emerged on the plates, which were transferred onto a new agar medium to obtain pure isolates. One of these isolates, coded as C5-9, was identified as a member of the genus *Micrococcus* on the basis of 99.9% similarity in the 16S rRNA gene sequence (1395 nucleotides; DNA Data Bank of Japan/DDBJ accession number LC498624) to *Micrococcus yunnanensis* YIM 65004^T^ (accession number FJ214355).

### Fermentation

Strain C5-9 was maintained on Marine Agar 2216 (Difco). A loopful of strain C5-9 was inoculated into a 500 mL K-1 flask containing 100 mL of Marine Broth 2216 (Difco) as a seed culture. The seed culture was incubated at 30 °C on a rotary shaker at 200 rpm for two days. Three mL each of the seed culture were inoculated into 29 500 mL K-1 flasks containing 100 mL of A16 production medium, which consisted of glucose 2%, Pharmamedia (Traders Protein, Memphis, TN, USA) 1%, CaCO_3_ 0.5%, Diaion HP-20 (Mitsubishi Chemical, Kanagawa, Japan) 1%, and natural seawater collected in Toyama Bay, Toyama, Japan. The pH value of the medium was adjusted to 7.0 before sterilization. The inoculated flasks were incubated at 30 °C for 5 days with rotational shaking using a rotary shaker at a speed of 200 rpm.

### Extraction and isolation

After fermentation, 100 mL of 1-butanol were added to each flask, and the flasks were shaken for 1 h. The emulsified mixture was centrifuged at 6000 rpm for 10 min, and the organic layer was separated from the aqueous layer. The organic layer was concentrated in vacuo to afford 5.1 g of crude extract from 2.9 L of production culture. The extract was successively partitioned between 60% aqueous MeOH (500 mL) and CH_2_Cl_2_ (500 mL × 3) and the latter between 90% aqueous MeOH (250 mL) and *n*-hexane (250 mL × 3). The 90% aqueous MeOH layer was evaporated to dryness (577 mg) and then fractionated by ODS column chromatography with a gradient of MeCN–0.1% HCO_2_H in aqueous solution (2:8, 3:7, 4:6, 5:5, 6:4, 7:3, and 8:2, v/v). Fraction 5 (6:4) was concentrated in vacuo, and the remaining aqueous layer was extracted with EtOAc. The organic layer was dried over anhydrous Na_2_SO_4_, filtered, and concentrated to give 27.5 mg of semipure material. Final purification was achieved by preparative HPLC (Cosmosil Cholester, Nacalai Tesque Inc., 10 × 250 mm, 4 mL/min, UV detection at 290 nm) with an isocratic elution of MeCN/0.1% HCO_2_H (63:37) to afford **1** (3.7 mg, *t*_R_ 14.2 min) and **2** (2.4 mg, *t*_R_ 15.7 min).

(6*E*,8*Z*)-5-Oxo-6,8-tetradecadienoic acid (**1**): pale yellow amorphous solid; UV (MeOH) λ_max_ (log ε) 277 nm (4.25); IR (ATR) ν_max_: 3050, 2955, 1708, 1689 cm^−1^; HRESIMS-TOF (*m*/*z*): [M + Na]^+^ calcd for C_14_H_22_O_3_Na, 261.1461; found, 261.1453

(6*E*,8*E*)-5-Oxo-6,8-tetradecadienoic acid (**2**): pale yellow amorphous solid; UV (MeOH) λ_max_ (log ε) 275 nm (4.30); IR (ATR) ν_max_: 3389, 2929, 1710, 1659 cm^−1^; HRESIMS-TOF (*m*/*z*): [M + Na]^+^ calcd for C_14_H_22_O_3_Na, 261.1461; found, 261.1458.

### NMR spin system simulation

In order to determine the multiplicity pattern and the coupling constants for the double bond system of **2**, spin system simulations were performed using the freeware nmrpeak.exe [19].

### Antimicrobial assay

Antimicrobial assays were carried out in a similar manner as described in [18]. The antimicrobial activity was evaluated by the liquid microculture method using round-bottomed 96-well microtiter plates against five bacteria, *Micrococcus luteus* ATCC9341, *Staphylococcus aureus* FDA209P JC-1, *Rhizobium radiobacter* NBRC14554, *Escherichia coli* NIHJ JC-2, *Tenacibaculum maritimum* NBRC16015, and two yeasts, *Candida albicans* NBRC0197 and *Saccharomyces cerevisiae* S100, as indication strains. Mueller–Hinton Broth (Difco), Sabouraud Dextrose Broth (Difco), and Marine Broth (Difco) were used for bacteria, yeasts, and *Tenacibaculum maritimum* NBRC16015, respectively. Compounds **1** and **2**, the reference drugs kanamycin sulfate for bacteria, sulfamethoxazole for *R. radiobacter* NBRC14554 and *T. maritimum* NBRC16015, and amphotericin B for yeasts, were made in twofold dilution series along the longer side of the plates by sequential transfer of 100 µL aliquots between the adjacent wells to which the same amount of medium was predispensed. To each well was added a 100 µL suspension of the indication strains prepared at ≈10^6^ cfu/mL from a culture at the logarithmic growth phase. The solvent vehicle added to the top rows was set at 0.5% of the final culture volume to avoid the effect on the growth of microbes. The plates were incubated at 37 °C for 20 h for bacteria, at 24 °C for *T. maritimum* NBRC16015, and at 32 °C for yeasts. The tests were done in triplicates, and the absorbance at a wavelength of 650 nm was measured with the help of a microplate reader.

### Cytotoxicity assay

The cytotoxicity assay was carried out in a similar manner as described in [17]. P388 murine leukemia cells were maintained in RPMI-1640 medium containing ʟ-glutamine (product no. 186-02155) supplemented with 10% fetal bovine serum and 0.1 mg/mL gentamicin sulfate. Compounds **1**, **2**, and doxorubicin as a reference were serially diluted by a factor of 3.16 (half-logarithmic dilution) in a 96-well round-bottom microtiter plate. To each well were seeded the cells at a final density of 5 × 10^3^ cells/well, and 200 µL cultures thus made were incubated for 96 h at 37 °C in an atmosphere of 5% CO_2_ in air with 100% humidity. The viability of the cells was visualized by the addition of 50 μL of the medium containing XTT (1 mg/mL) and PMS (40 µg/mL) to each well. After incubating for 4 h at 37 °C, the medium was carefully removed by a suction aspirator, and formazan dye, formed by respiratory reduction by living cells, was quantified by the absorption at 450 nm, read by a microplate reader to calculate the rate of cell growth inhibition at each concentration, and the results of the triplicates were plotted on single-logarithmic charts to deduce the IC_50_ values.

### PPAR activation assay

Measurements of PPARα, -β/δ, and -γ ligand activity was evaluated by a luciferase reporter gene assay system [49]. Briefly, COS-1 cells (5 × 10^5^ cells) were transiently transfected with an expression plasmid containing the ligand-binding domain of human PPARα, -β/δ, and -γ fused to the GAL4 DNA-binding domain (pPPARα–GAL4, pPPARδ–GAL4, or pPPARγ–GAL4, 0.25 µg), a luciferase reporter plasmid 17m2G TATA Luc (p17m2G, 1 µg), and the pSEAP-control vector (1 µg, Clontech, CA, USA) by using the Effectene transfection reagent. Transfection was performed in 60 mm culture dishes according to the manufacturer’s instructions. After 16 h, the transfected cells were recovered and seeded into 96-well white multiwell plates, the indicated concentrations of the test compounds were added, and the plates were cultured for an additional 24 h at 37 °C in a 5% CO_2_ incubator. The luciferase activity and secreted alkaline phosphatase SEAP activity were measured in each well by using a Steady-Glo^®^ luciferase assay (Promega, Madison, WI, USA) and Great ESCAPe SEAP Reporter System3 (Clontech), according to the manufacturer’s instructions. The SEAP activity level was used to correct the luciferase activity in each well. Each data value is presented as the mean ± standard error of three experiments.

## Supporting Information

File 1 ESIMS-TOF, UV, IR, 1D, and 2D NMR spectra of **1** and **2**.
