# Supplementary material for: Two antibacterial and PPARα/γ-agonistic unsaturated keto fatty acids from a coral-associated actinomycete of the genus Micrococcus
Source: Beilstein J Org Chem. 2020 Mar 2;16:297–304. doi: 10.3762/bjoc.16.29 (PMC7082699; doi:10.3762/bjoc.16.29)
Supplement: File 1 — ESIMS-TOF, UV, IR, 1D, and 2D NMR spectra of 1 and 2. [file Beilstein_J_Org_Chem-16-297-s001.pdf]

BEILSTEIN JOURNAL OF ORGANIC CHEMISTRY

Supporting Information

for

Two antibacterial and PPARα/γ-agonistic unsaturated keto fatty acids from a coral-associated actinomycete of the genus *Micrococcus*

Amit Raj Sharma, Enjuro Harunari, Naoya Oku, Nobuyasu Matsuura, Agus Trianto and Yasuhiro Igarashi

Beilstein J. Org. Chem. **2020,** *16,* 297–304. doi:10.3762/bjoc.16.29

ESIMS-TOF, UV, IR, 1D, and 2D NMR spectra of 1 and 2

License and Terms: This is a supporting information file under the terms of the Creative Commons Attribution License (https://creativecommons.org/licenses/by/4.0). Please note that the reuse, redistribution and reproduction in particular requires that the authors and source are credited.
The license is subject to the *Beilstein Journal of Organic Chemistry* terms and conditions: (https://www.beilstein-journals.org/bjoc)

Table of contents

Figure S1: High resolution ESI-TOF mass spectra of (6*E*,8*Z*)-5-oxo-6,8-tetradecadienoic acid (**1**) and (6*E*,8*E*)-5-oxo-6,8-tetradecadienoic acid (**2**)

Figure S2: UV spectra of **1** and **2**

Figure S3: IR spectra of **1** and **2**

Figure S4: ^{1}H NMR spectrum of **1** (500 MHz, $CDCl_3$)

Figure S5: ^{13}C NMR spectrum of **1** (125 MHz, $CDCl_3$)

Figure S6: DEPT135 spectrum of **1** (125 MHz, $CDCl_3$)

Figure S7: COSY spectrum of **1** (500 MHz, $CDCl_3$)

Figure S8: HSQC spectrum of **1** (500 MHz, $CDCl_3$)

Figure S9: HMBC spectrum of **1** (500 MHz, $CDCl_3$)

Figure S10: ^{1}H NMR spectrum of **2** (500 MHz, $CDCl_3$)

Figure S11: ^{13}C NMR spectrum of **2** (125 MHz, $CDCl_3$)

Figure S12: DEPT135 spectrum of **2** (125 MHz, $CDCl_3$)

Figure S13: COSY spectrum of **2** (500 MHz, $CDCl_3$)

Figure S14: HSQC spectrum of **2** (500 MHz, $CDCl_3$)

Figure S15: HMBC spectrum of **2** (500 MHz, $CDCl_3$)

(a)

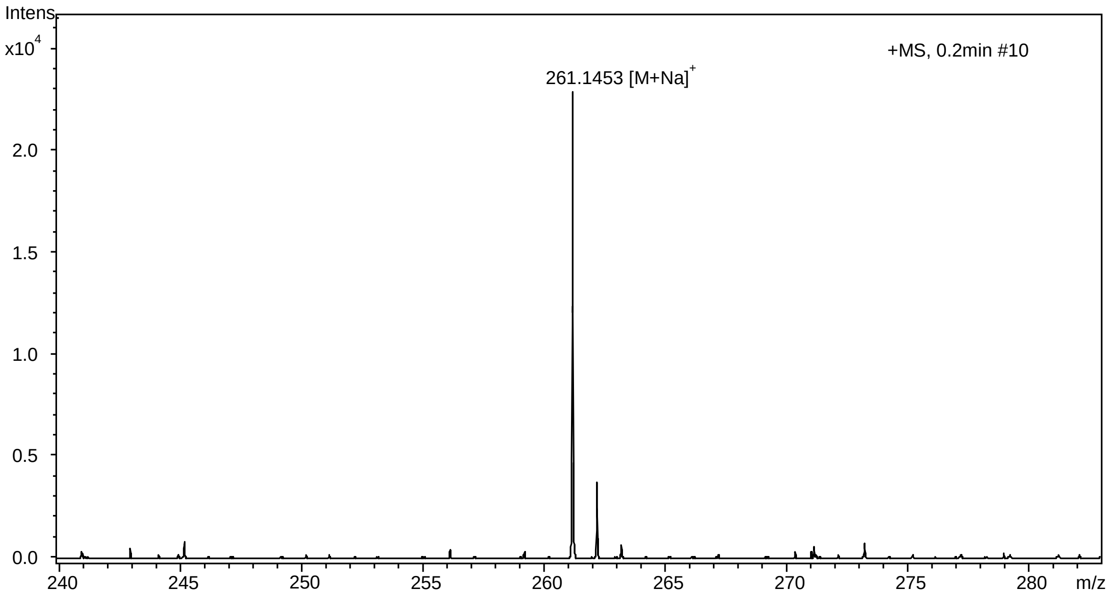

(b)

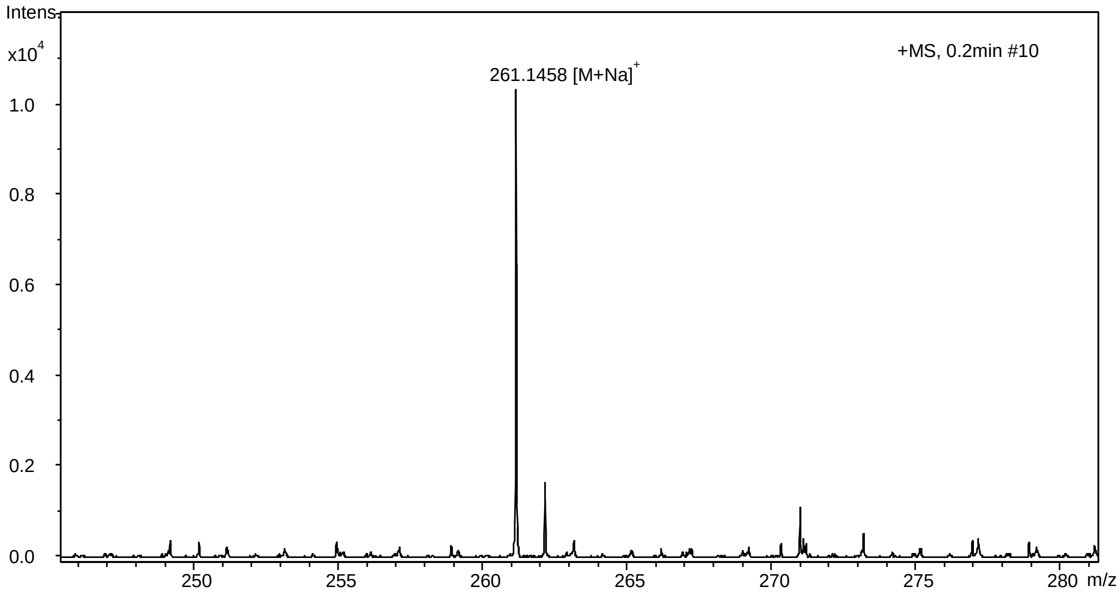

Figure S1: High resolution ESI-TOF mass spectra of (a) (6*E*,8*Z*)-5-oxo-6,8-tetradecadienoic acid (**1**) and (b) (6*E*,8*E*)-5-oxo-6,8-tetradecadienoic acid (**2**).

(a) (**1)** (MeOH)

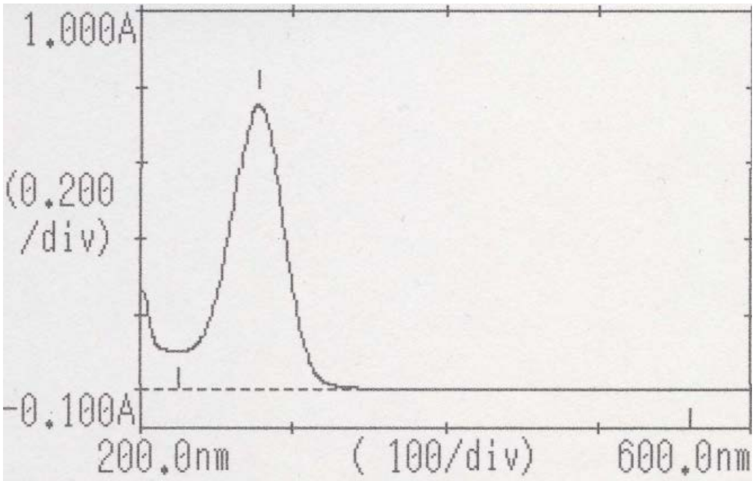

(b) (**2**) MeOH

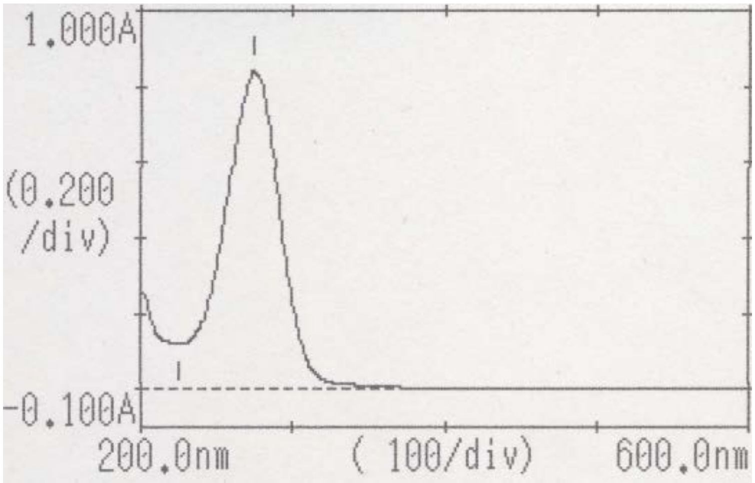

Figure S2: UV spectra of **1** and **2**.

(a) **1** (ATR)

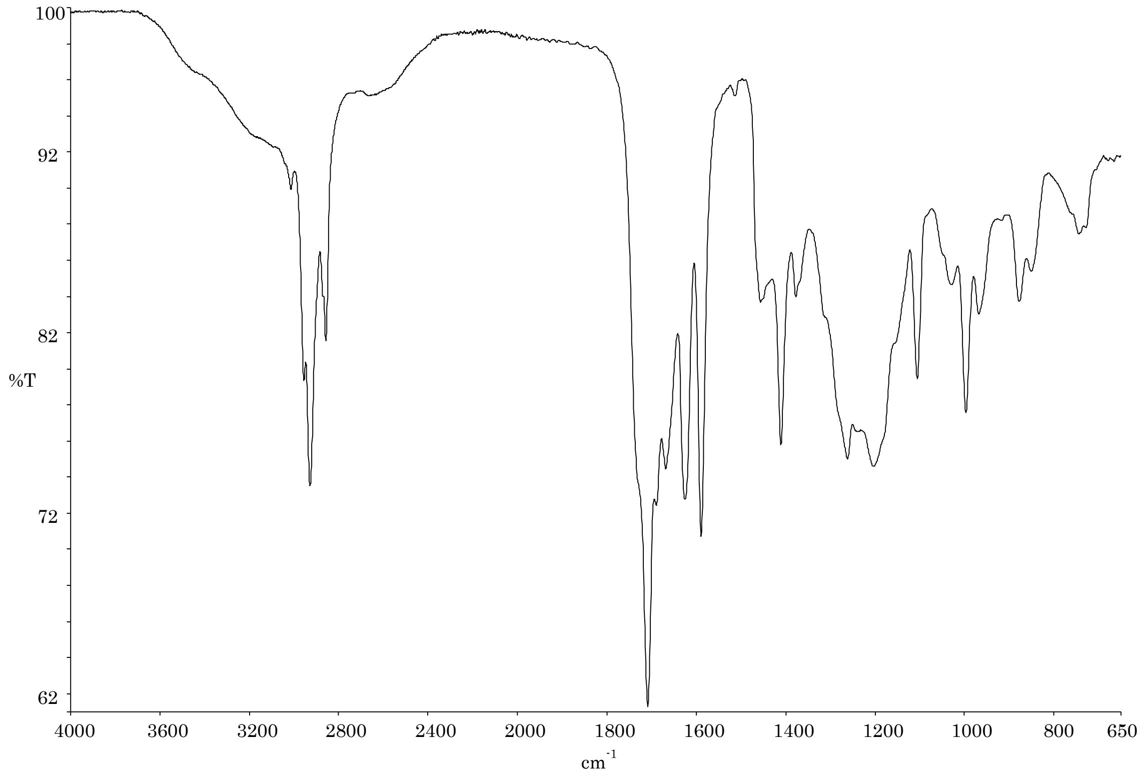

(b) **2** (ATR)

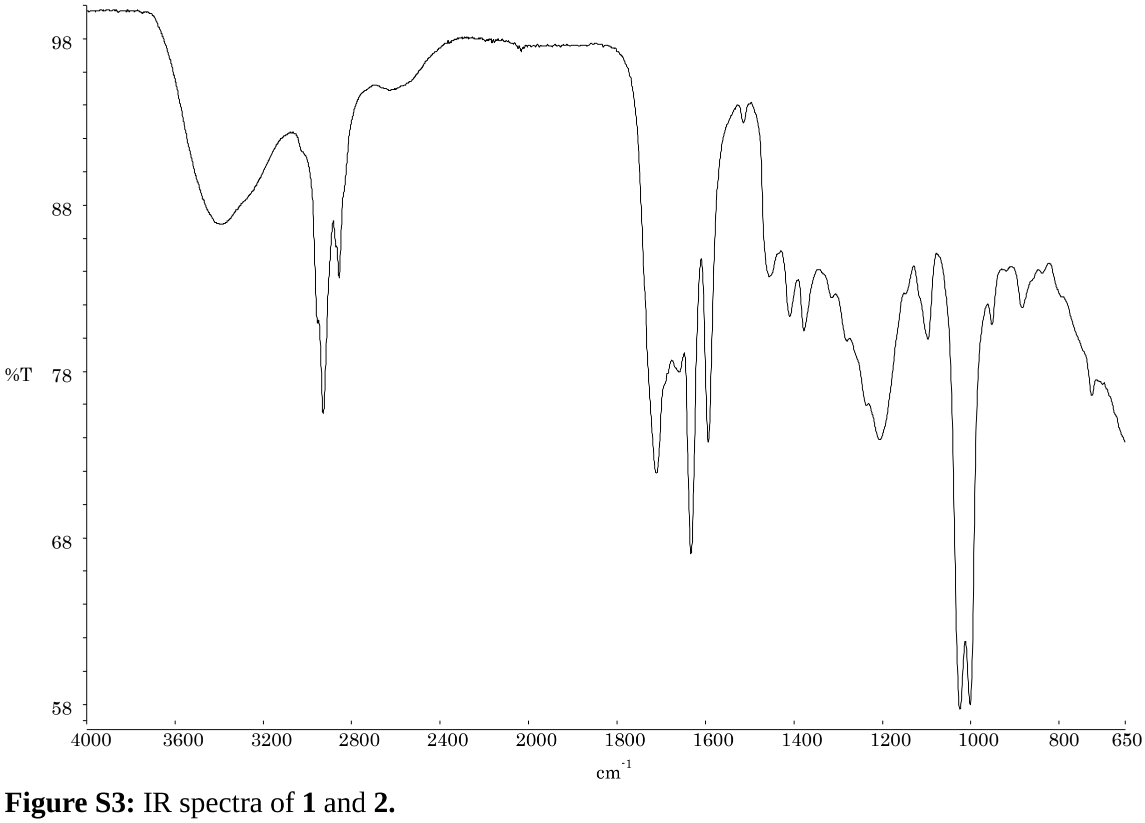

Figure S3: IR spectra of **1** and **2.**

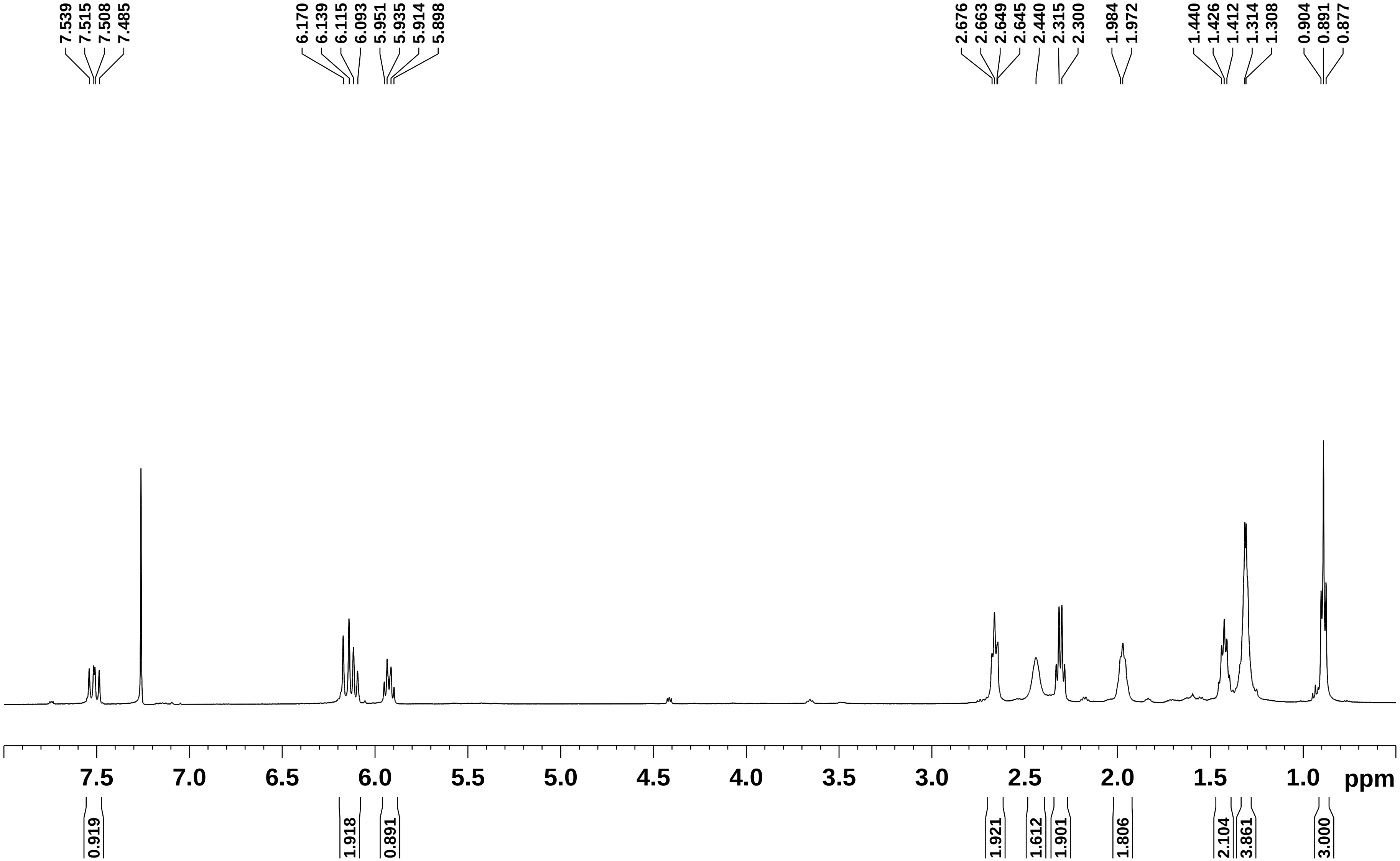

Figure S4: ^{1}H NMR spectrum of **1** (500 MHz, $CDCl_3$).

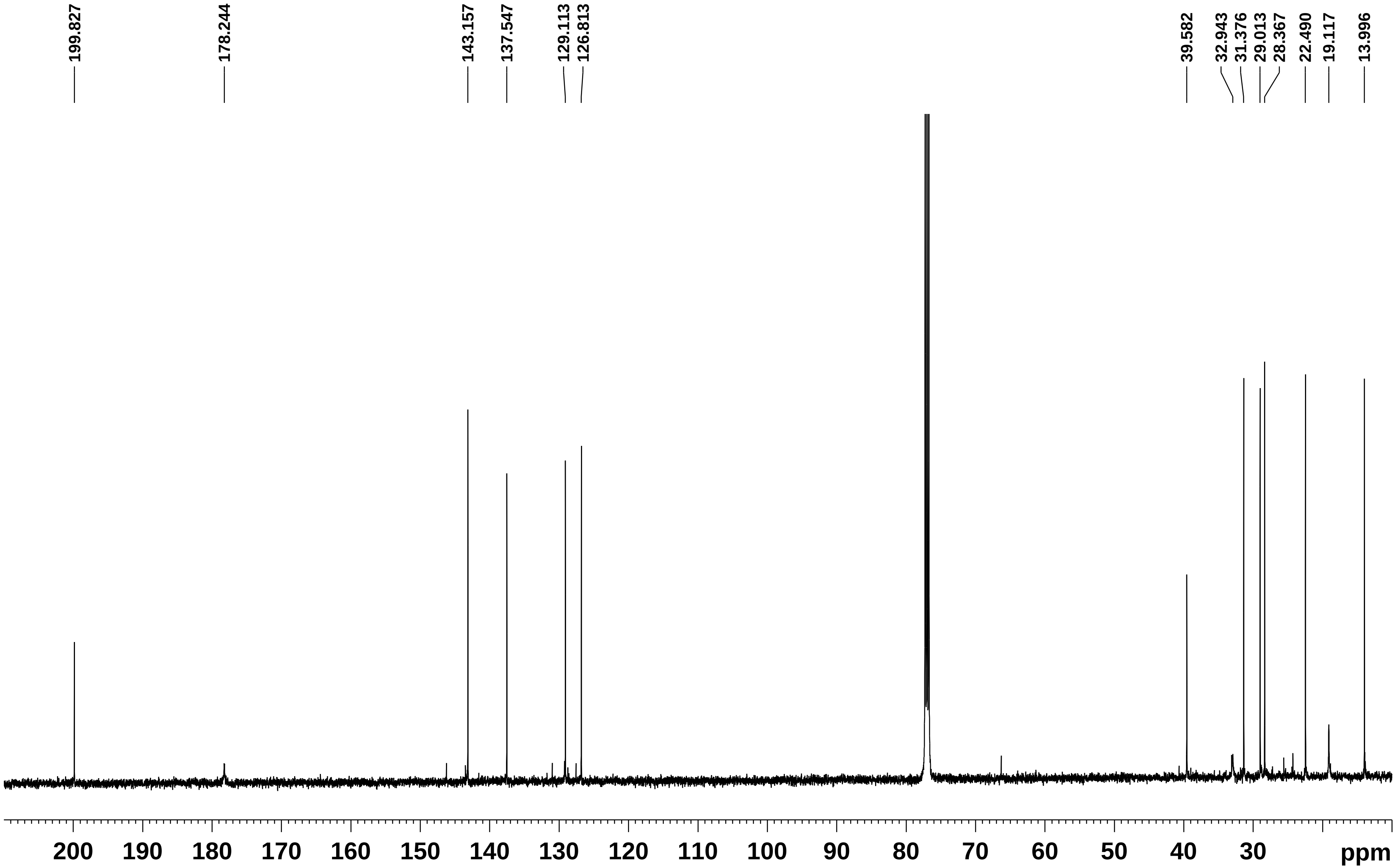

Figure S5: ^{13}C NMR spectrum of **1** (125 MHz, $CDCl_3$).

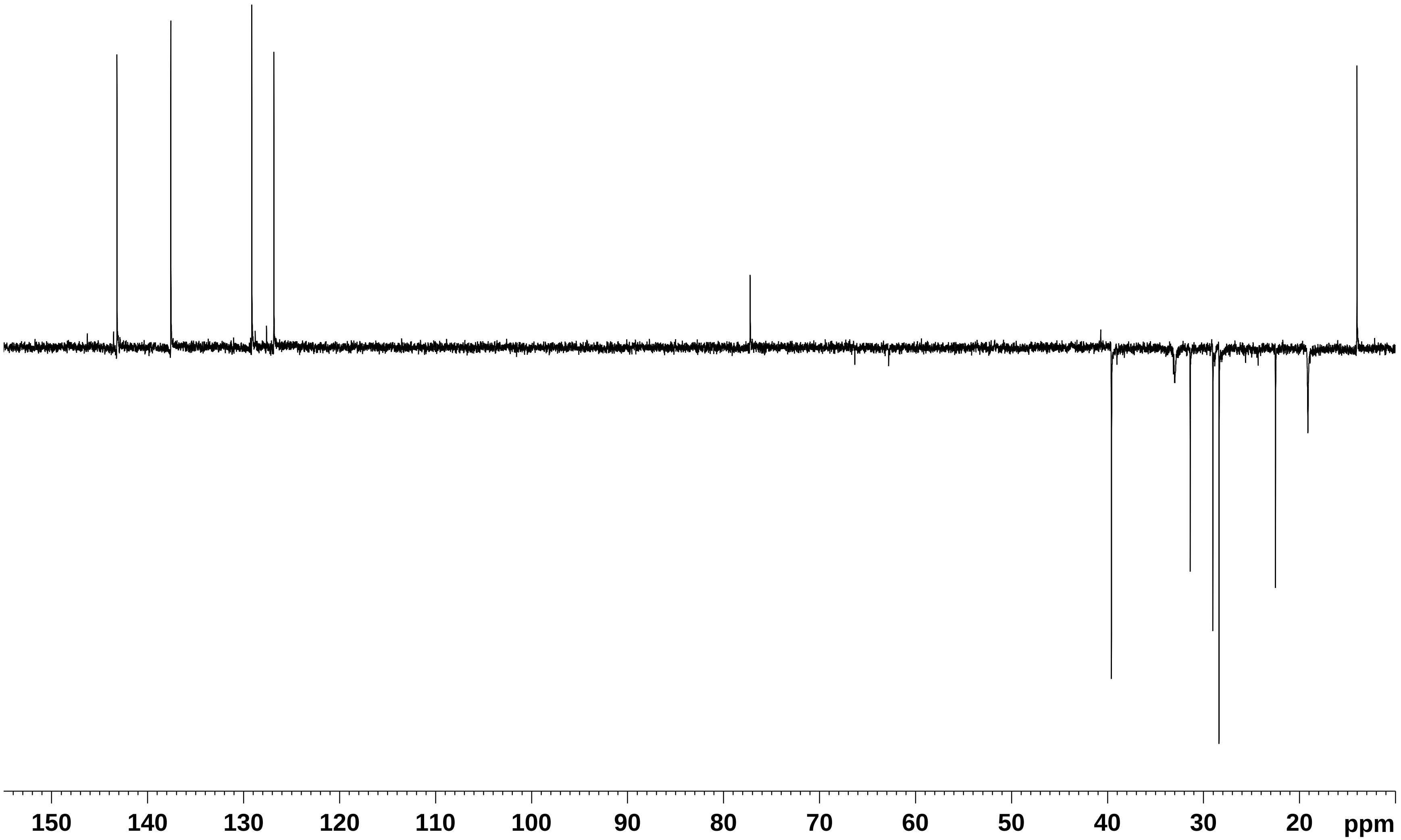

Figure S6: DEPT135 spectrum of **1** (125 MHz, $CDCl_3$).

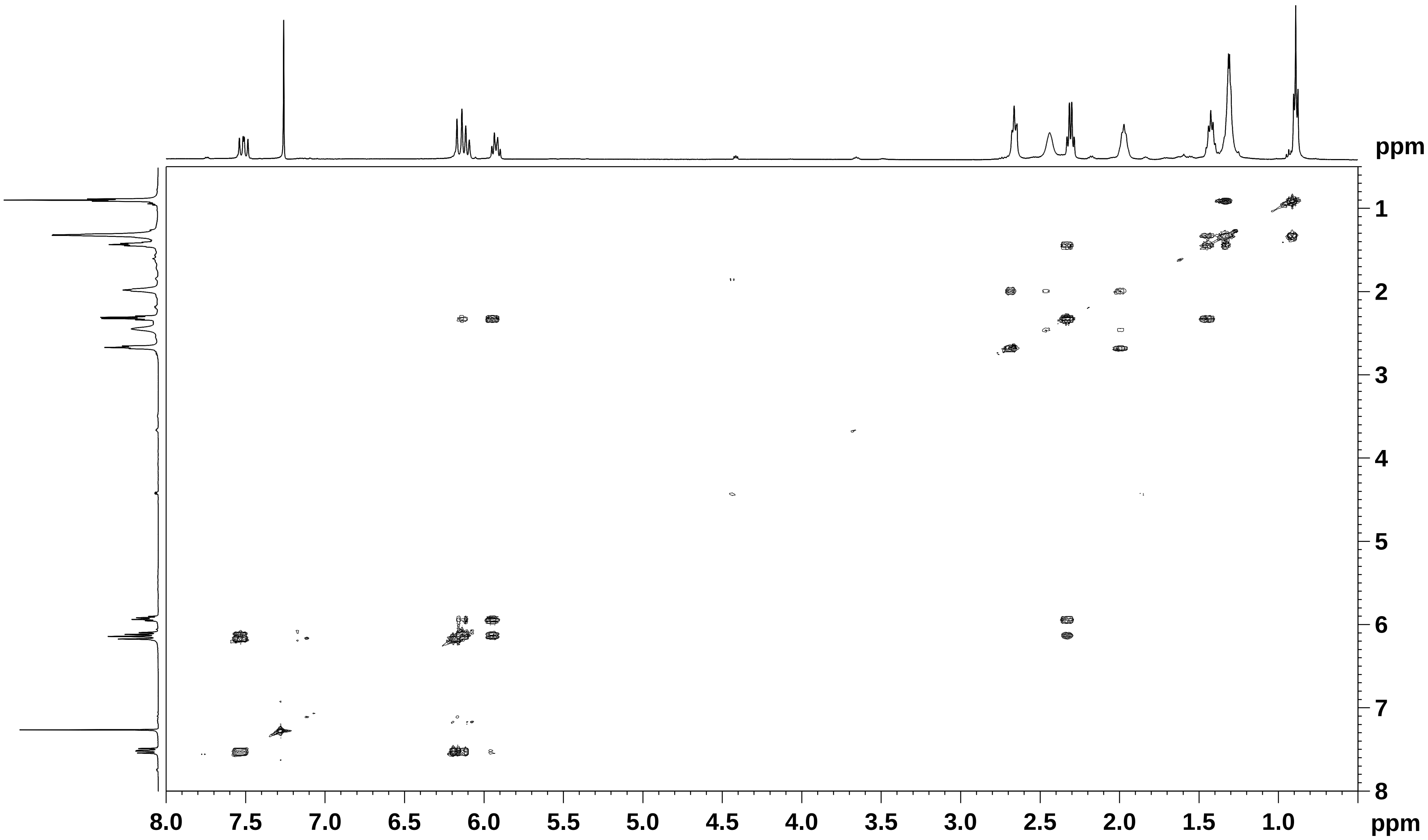

Figure S7: COSY spectrum of **1** (500 MHz, $CDCl_3$).

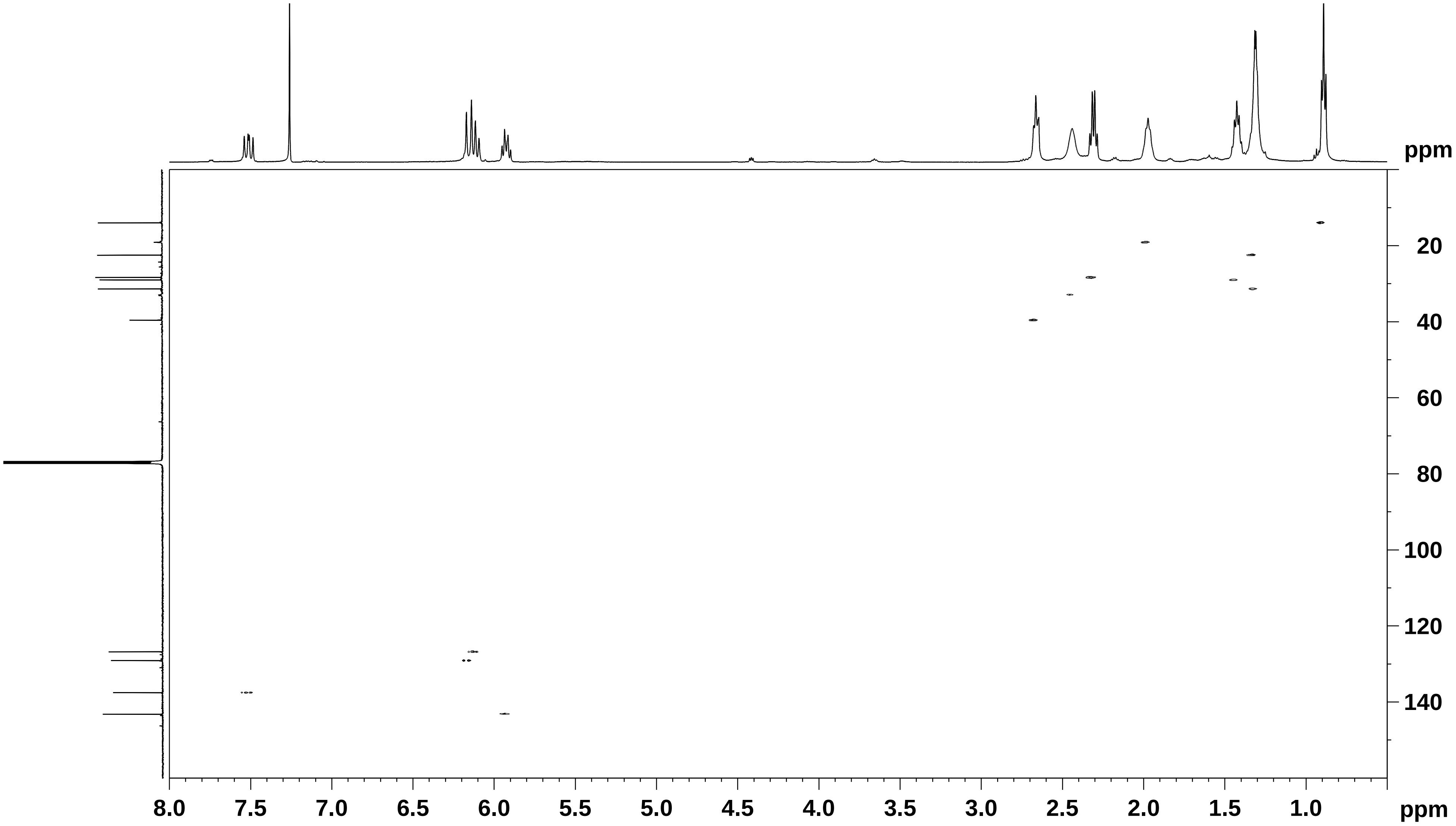

Figure S8: HSQC spectrum of **1** (500 MHz, $CDCl_3$).

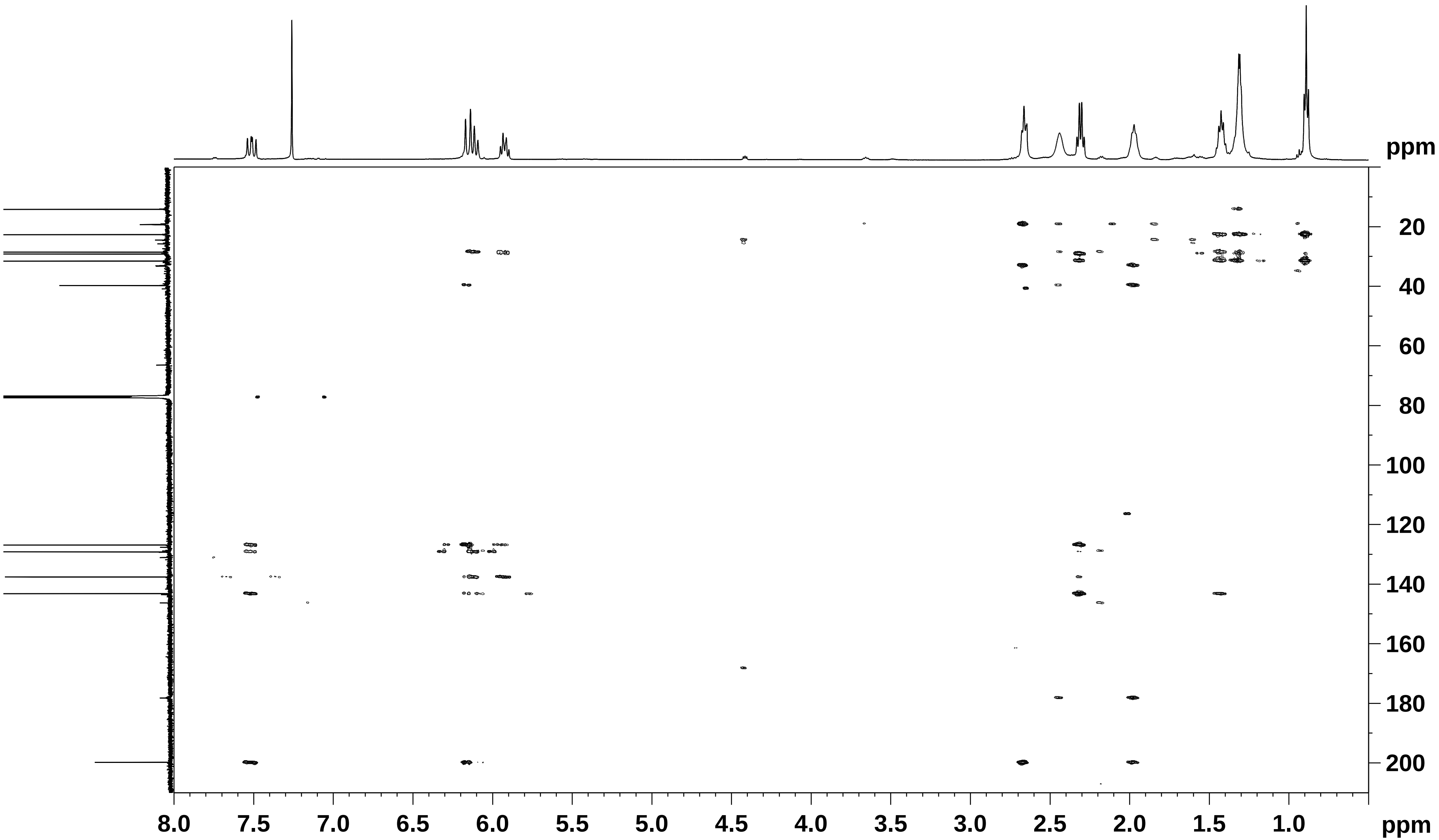

Figure S9: HMBC spectrum of **1** (500 MHz, $CDCl_3$).

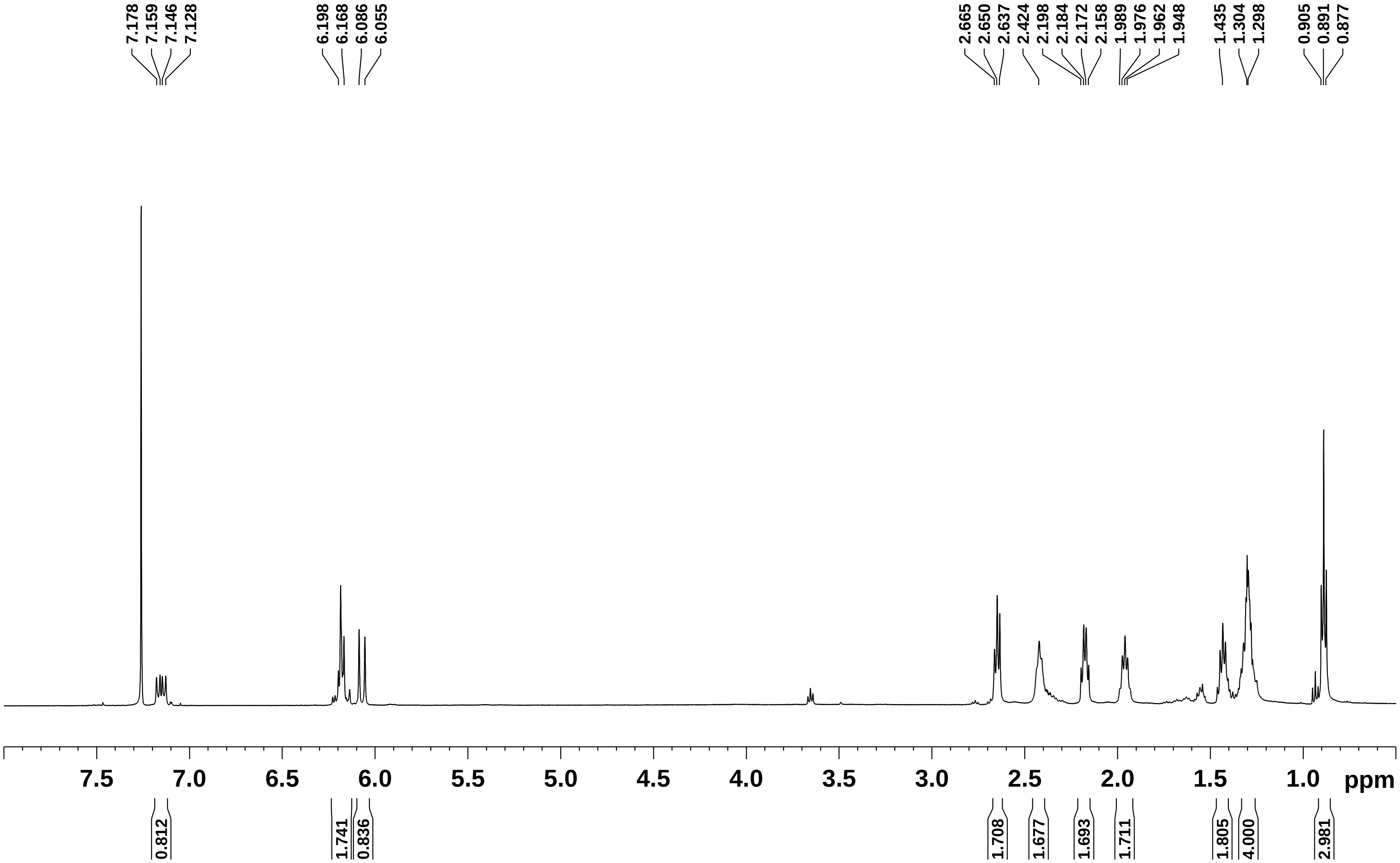

Figure S10: ^{1}H NMR spectrum of **2** (500 MHz, $CDCl_3$).

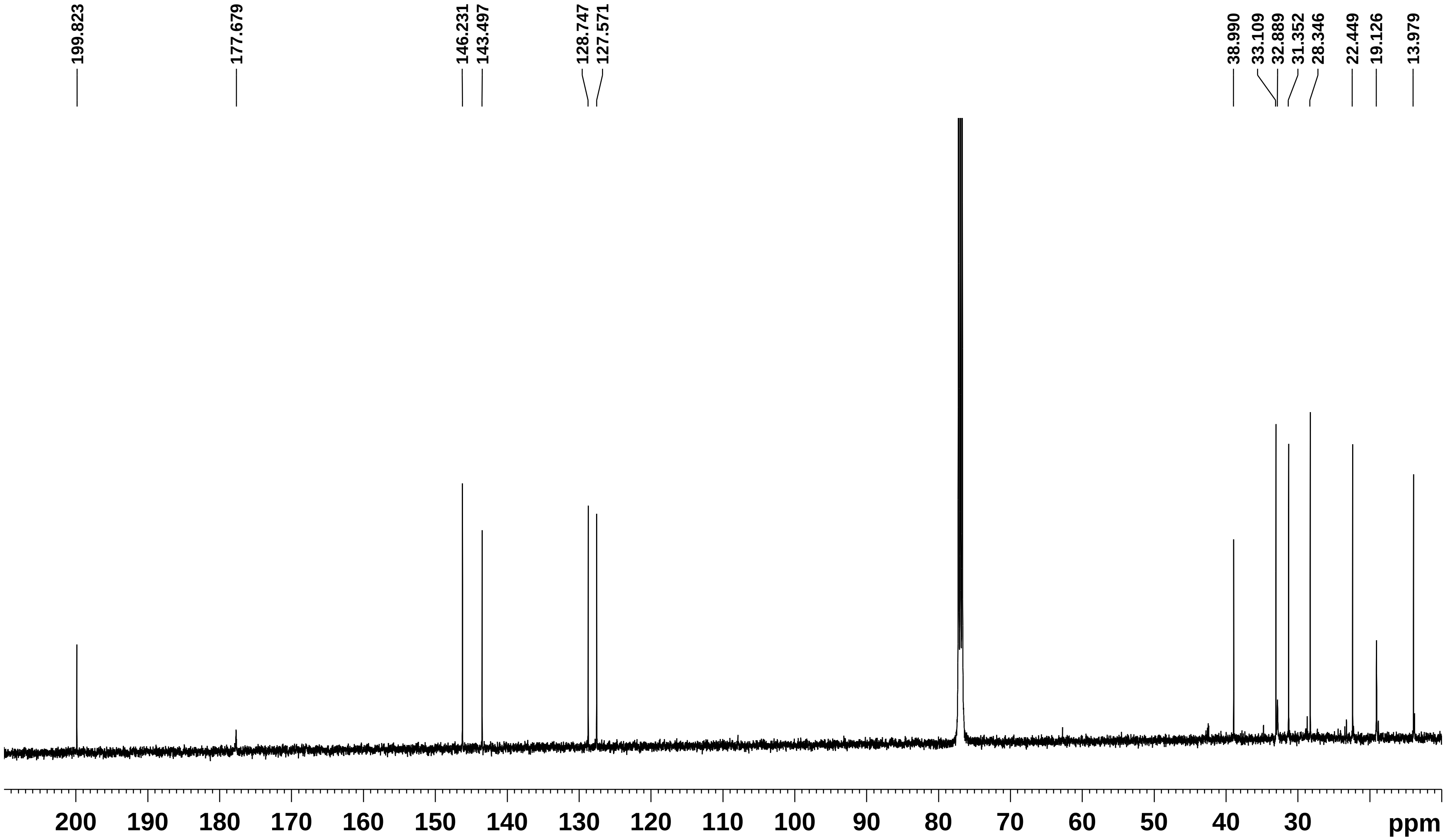

Figure S11: ^{13}C NMR spectrum of **2** (125 MHz, $CDCl_3$).

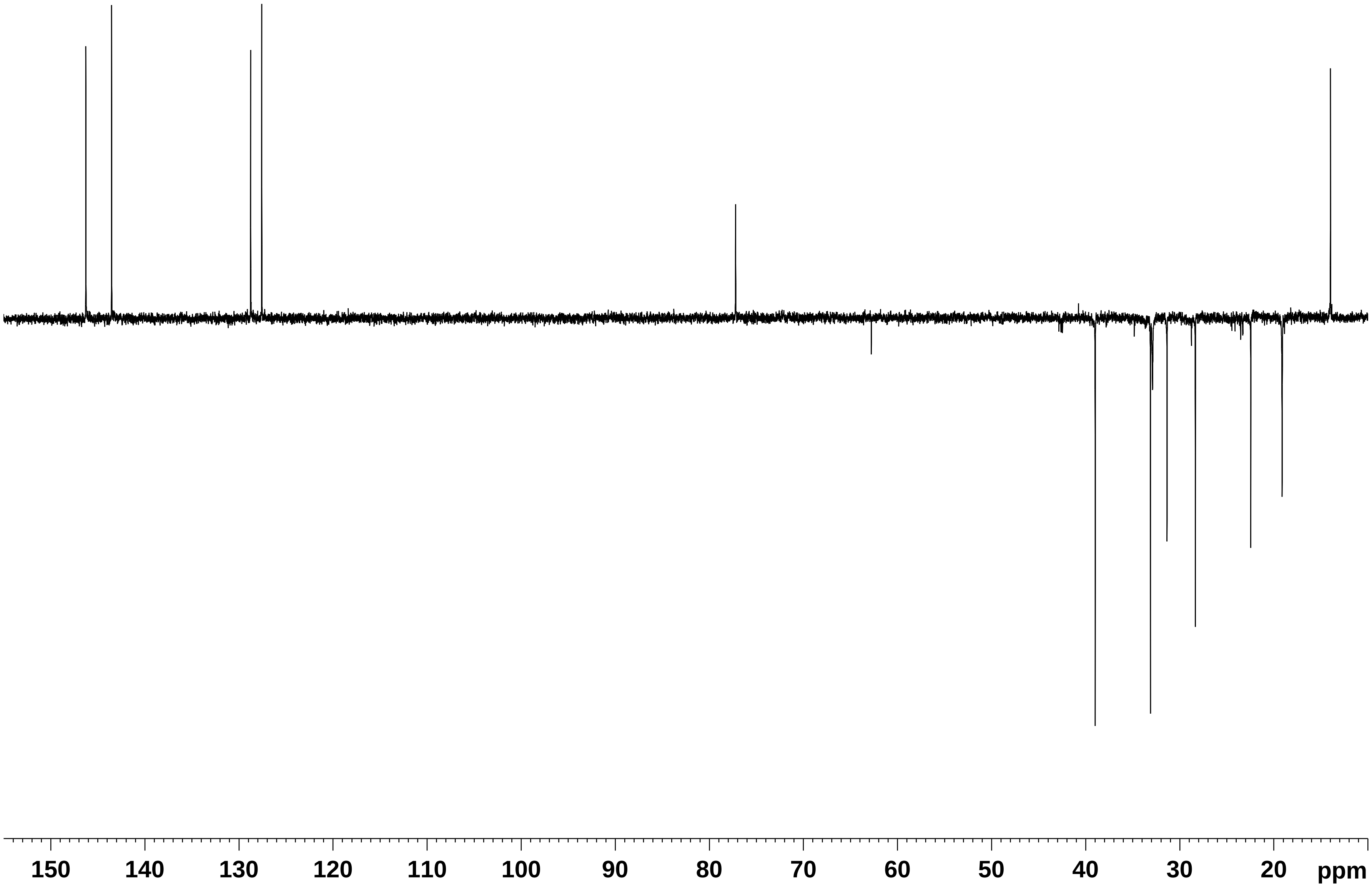

Figure S12: DEPT135 spectrum of **2** (125 MHz, $CDCl_3$).

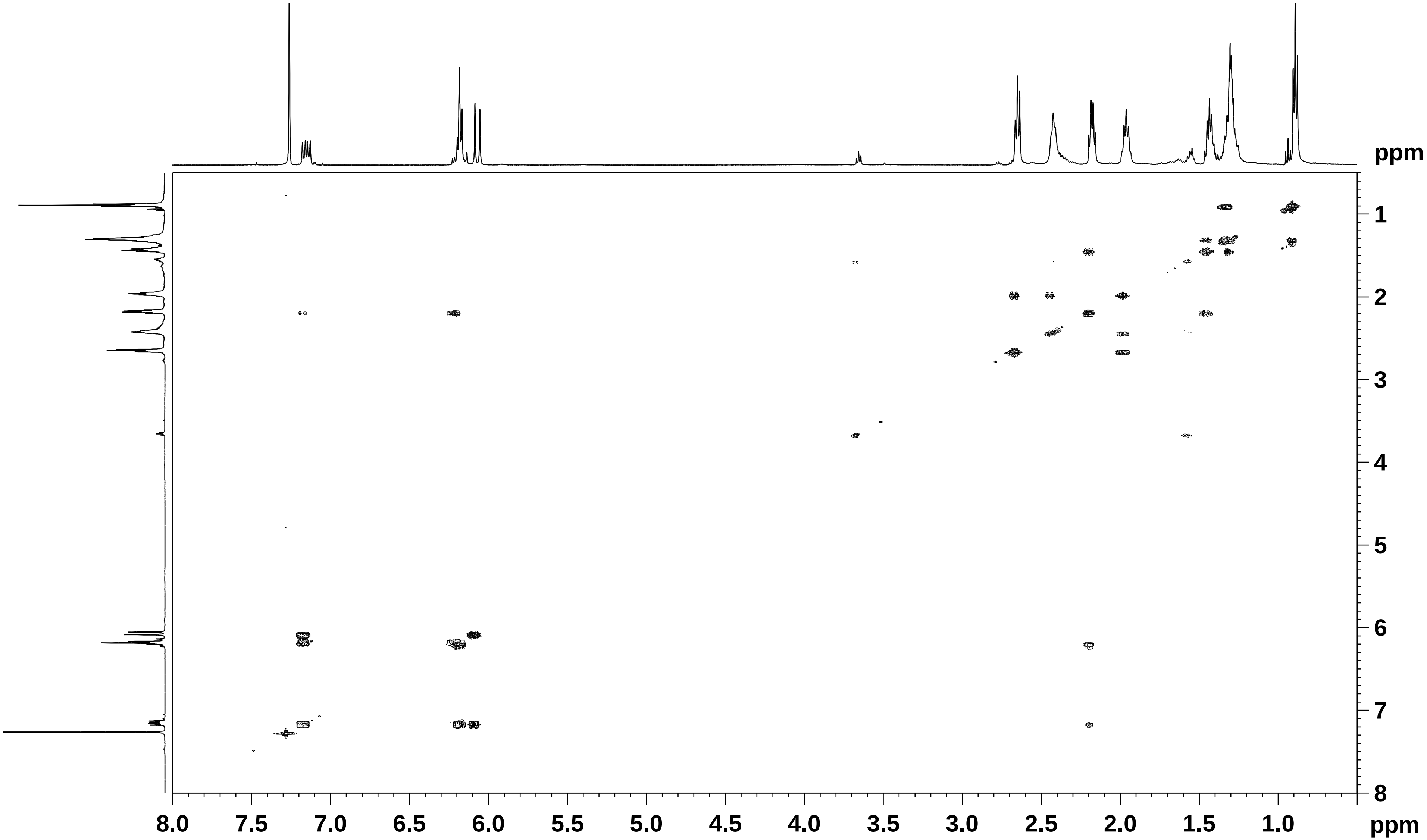

Figure S13: COSY spectrum of **2** (500 MHz, $CDCl_3$).

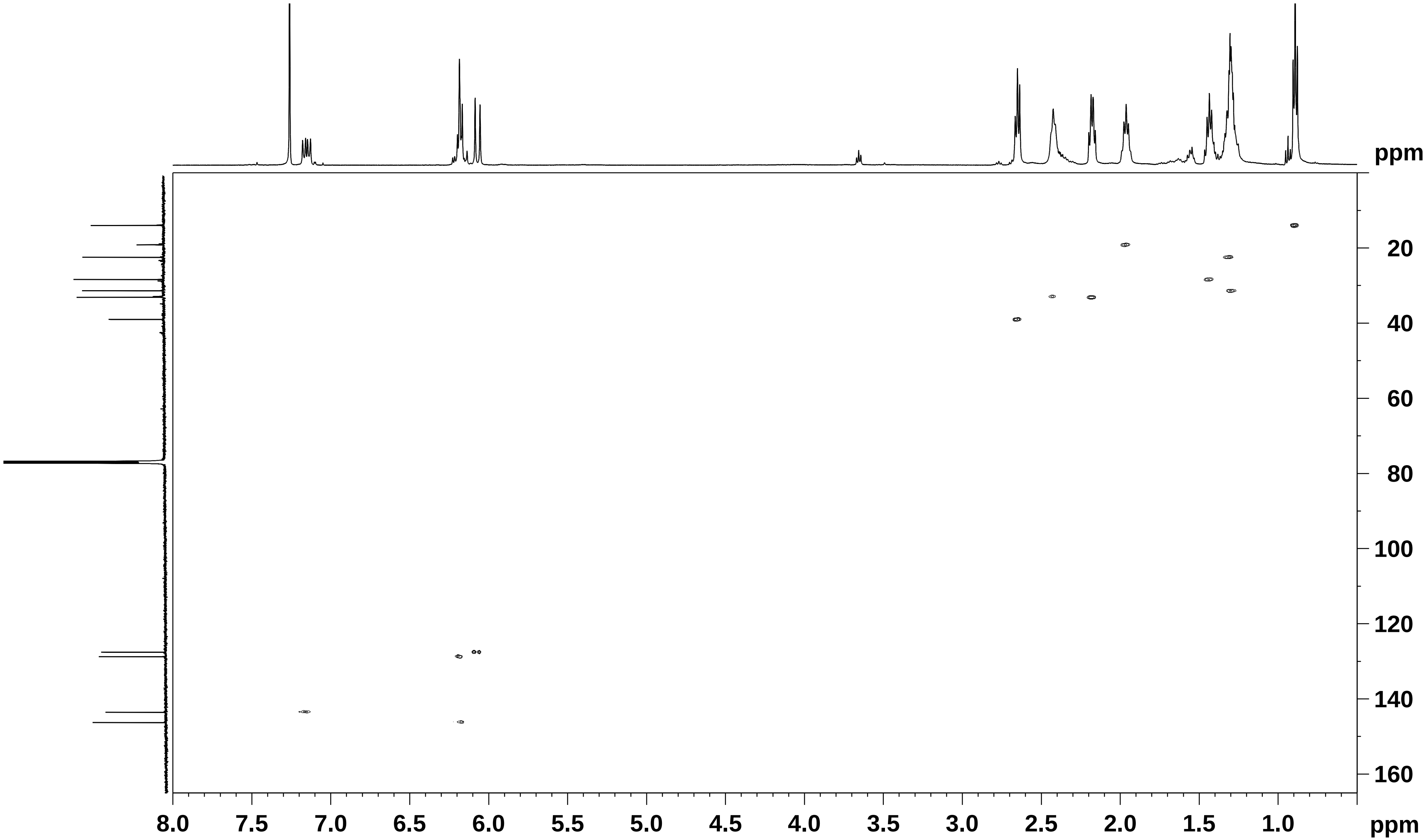

Figure S14: HSQC spectrum of **2** (500 MHz, $CDCl_3$).

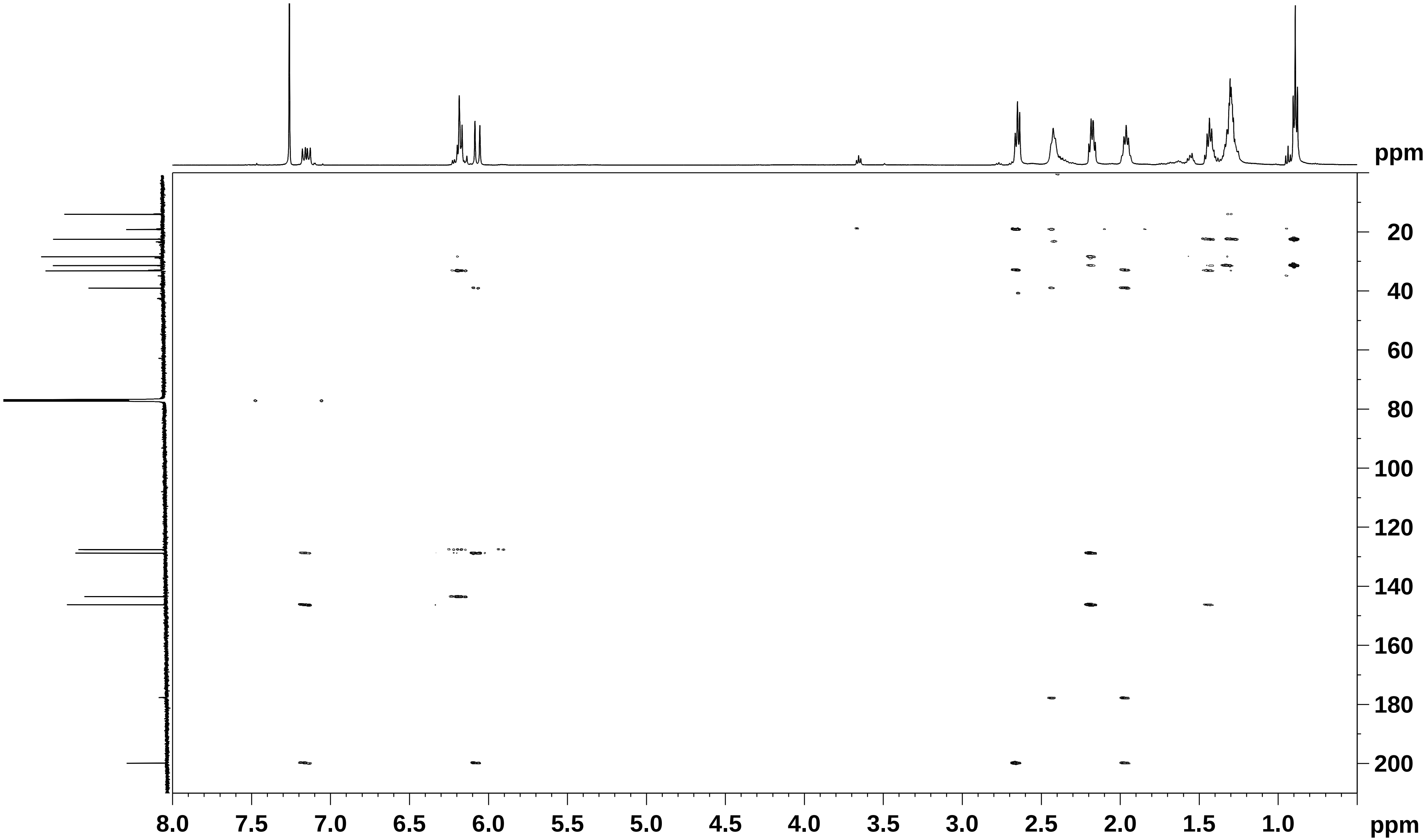

Figure S15: HMBC spectrum of **2** (500 MHz, $CDCl_3$).
